# Biodistribution of unmodified cardiosphere‐derived cell extracellular vesicles using single RNA tracing

**DOI:** 10.1002/jev2.12178

**Published:** 2022-01-10

**Authors:** Alessandra Ciullo, Chang Li, Liang Li, Korie C. Ungerleider, Kiel Peck, Eduardo Marbán, Ahmed G.E. Ibrahim

**Affiliations:** ^1^ Smidt Heart Institute Cedars‐Sinai Medical Center Los Angeles California USA

**Keywords:** biodistribution, extracellular vesicles, qPCR, small non‐coding RNA, Y‐derived small RNA cardiosphere‐derived cells, YsRNA

## Abstract

Extracellular vesicles (EVs) are potent signalling mediators. Although interest in EV translation is ever‐increasing, development efforts are hampered by the inability to reliably assess the uptake of EVs and their RNA cargo. Here, we establish a novel qPCR‐based method for the detection of unmodified EVS using an RNA Tracer (DUST). In this proof‐of‐concept study we use a human‐specific Y RNA‐derived small RNA (YsRNA) we dub “NT4” that is enriched in cardiosphere‐derived cell small EVs (CDC‐sEVs). The assay is robust, sensitive, and reproducible. Intravenously administered CDC‐sEVs accumulated primarily in the heart on a per mg basis. Cardiac injury enhanced EV uptake in the heart, liver, and brain. Inhibition of EV docking by heparin suppressed uptake variably, while inhibition of endocytosis attenuated uptake in all organs. In vitro, EVs were uptaken more efficiently by macrophages, endothelial cells, and cardiac fibroblasts compared to cardiomyocytes. These findings demonstrate the utility of DUST to assess uptake of EVs in vivo and in vitro.

## 1 INTRODUCTION

Extracellular vesicles (EVs) comprise several classes of lipid bilayer particles that derive either from the late endosome or are shed passively from the plasma membrane. EVs play pivotal roles in diseases including cancer and ischemic injury and, as recognized more recently, mediate the regenerative effects of cell therapy. EVs contain potent bioactive cargo including small RNAs, proteins, and lipids which alter the transcriptome and epigenome of recipient tissue. Cardiosphere‐Derived Cells (CDCs) are a population of heart stromal progenitors with immunomodulatory, anti‐fibrotic, angiogenic, and tissue‐reparative properties (De Couto et al., 2015; Kreke et al., 2012; Makkar et al., 2012; Marbán, 2014; Smith et al., 2007). CDCs secrete small EVs (CDC‐sEVs) (Cambier et al., 2017; Gallet et al., 2017; Ibrahim et al., 2014) which are necessary, and sufficient, to rationalize the cells’ benefits. As such, CDC‐sEVs represent attractive next‐generation cell‐free therapeutics. Indeed, EVs circumvent key challenges inherent to cell therapy. As non‐living agents, they do not require the scale‐up and handling measures that cells require to preserve viability (Zhang et al., 2014). EVs are also less immunogenic than cells (Marbán, 2014), which, coupled with their small size, allow for larger and repeated doses (Lai et al., 2012).

One of the enduring challenges in EV biology is tracking the biodistribution of RNA cargo reliably in vivo. Understanding EV cargo biodistribution is critical to understanding pharmacokinetics and advancing clinical translation. Lipophilic dyes are fraught with challenges including introducing a lack of specificity (Takov et al., 2017). EV marker‐reporter manipulation is limited only to surface markers and introduces marker exclusivity (Koliha et al., 2016) as well as the inability to mark biological fluids such as serum. Finally, EV tracking methods fall short of confirming delivery of cargo which is particularly useful in signalling and translational application. RNA comprises one of the most bioactive signalling mediators in EVs. Thus, assessing the biodistribution of this key class of EV cargo is useful in studying EV signal transduction (Veziroglu & Mias, 2020). Here we have developed a novel qPCR‐based method we call Detection of Unmodified EVS using RNA Tracer (DUST) for measuring biodistribution and retention of RNA cargo from unaltered EVs. By identifying a naturally abundant small RNA species with low background in mouse tissue, we establish a proof of concept for an approach that can be adapted to other unmanipulated EV populations. We use DUST to evaluate the biodistribution of CDC‐sEVs in both healthy animals and those with ischemia‐reperfusion injury for which this EV therapy is indicated.

## 2 METHODS

### 2.1 CDC cell culture

CDCs were prepared as described previously (Smith et al., 2007). Briefly, atria and ventricular septum were obtained from the healthy hearts of deceased tissue donors. Tissue was chopped, mixed in 1:4 atria to septum ratio, washed, and seeded on CellBIND flasks (Corning). Explants were incubated at 37°C, 5% CO_2_, 5% O_2_ in IMDM media supplemented with 20% FBS, for 2–3 weeks until outgrowth reached 80% confluence. Cells were then harvested using TrypLE Select (Thermo Fisher), filtered through a 100 μm Steriflip unit (Millipore) to remove explants, and resuspended in CryoStore CS10 (Stemcell Technologies) before freezing in liquid nitrogen. When needed, the frozen vial was removed from liquid nitrogen and seeded on Ultra‐Low Attachment flasks to form cardiospheres. CDCs were formed by seeding cardiospheres on fibronectin‐coated flasks and culturing at 37°C, 5% CO_2_, 5% O_2_ in IMDM supplemented with 10% FBS. Cells were conditioned at passage five or subjected to a second cardiosphere step and conditioned two passages after culturing on fibronectin‐coated plates.

### 2.2 Biodistribution

EVs preparation, isolation, and characterization. CDCs were conditioned in Iscove's Modified Dulbecco's Medium (IMDM) without supplementation for 15 days at 37°C and 20% O2. The conditioned media was collected, filtered through a 0.45 μm filter, and concentrated using 10 KDa Centricon Plus‐70 Centrifugal Filter (Millipore) or 1000 KDa Centricon Plus‐70 Centrifugal Filter (Millipore). Protein concentration was measured using the DC assay (Biorad). Particle size and concentration were measured on a NanoSight NS300 (Malvern). The parameters for Nanosight acquisition and analysis were as follows:

Camera level: 15

Detection Threshold: 5

Number of videos acquired per sample: 4

Video duration: 30 s

### 2.3 Cell and EVs lysates and protein assay

Cell and EV lysates were collected for western blot. Cells were pelleted and resuspended in 1× RIPA buffer (Pierce) with protease inhibitor on ice for 30 min. EVs resuspended in PBS were lysed in 10× RIPA buffer (Pierce) with protease inhibitor on ice for 30 min. Protein lysates were isolated by centrifugation at 14,000 rpm for 15 min at 4°C. Protein concentration was measured using a DC Protein Assay kit (Bio‐Rad).

### 2.4 Western blot

Membrane transfer was performed using the Turbo Transfer System (BIO‐RAD) after gel electrophoresis. The subsequent antibody staining was then applied and detected by SuperSignal West Pico PLUS Chemiluminescent Substrate (Thermo Fisher Scientific). Antibodies used in this study are the following: TSG101 (Invitrogen (MA1‐23296), CD81 (BD Biosciences # 555675), HSP90 (Abcam Ab13492), CD63 (Invitrogen # 106280), Calnexin (Cell Signaling, #2679S), Anti‐Rabbit IgG, HRP‐Linked Antibody (Cell Signaling Technology #7074), Anti‐Mouse IgG, HRP‐Linked Antibody (Cell Signaling Technology #7076).

### 2.5 Exo‐check exosome antibody array

Exosome protein expression was analyzed using an Exo‐Check Exosome Antibody Array (System Biosciences) using 300 μg of EV proteins per the manufacturer's protocol.

### 2.6 RNase and proteinase protection assay

For RNase and proteinase protection assay RNase A (Qiagen, 100 mg/ml stock solution) was used at a final concentration of 5 μg/ml, Proteinase K (Qiagen, 20 mg/ml stock solution) was used at a final concentration of 0.1 mg/ml and Triton X‐100 was used at 10% v/v. Briefly, 5 × 10^8^ CDC‐sEVs were treated with RNAse A for 20 min at 37°C. Samples were further treated with 0.1 mg/ml Proteinase K for 20 min at 37°C. Samples were pre‐treated with Triton 10% for 20 min at room temperature, followed by RNase A/Proteinase K incubation. EVs and RNA were isolated using the urine EV RNA isolation kit (Norgen Biotek, cat# 47200). qPCR was performed to assess NT4 expression levels. EVs purified using size‐exclusion chromatography (SEC, SmartSEC Single EV Isolation System,System biosciences) were used as further confirmation of findings.

#### 2.6.1 IMEX preparation

Immortalized CDCs were grown to confluence at 20% O_2_ at 37°C, and then cells were serum‐free at 2% O_2_ at 37°C overnight after three washes. Conditioned media was collected and filtered through a 0.45 μm filter to remove apoptotic bodies and cellular debris and EVs were purified using centrifugal ultrafiltration with a 10 KDa Centricon Plus‐70 Centrifugal Filter (Millipore). Particle size and concentration were measured using NanoSight NS300 (Malvern).

### 2.7 Cryo‐electron microscopy

For cryo‐EM, isolated CDC‐sEVs were resuspended in 30 mM HEPES, pH 7.4, containing 100 mM KCl. A 4–5 μl drop was deposited on glow‐discharged 200‐mesh copper grids with 2 μm holes (Quantifoil R2/2, Quantifoil Micro Tools), then plunge frozen in liquid ethane using a Leica EM‐GP (Leica Microsystems), at 90% humidity. For examination, all grids were transferred under liquid nitrogen to a Gatan 626 cryo‐holder (Gatan). Imaging was done under low‐dose conditions (∼10 e‐ per Å2) using a 4k×4x Ceta camera, on an FEI Talos 200C S/TEM (FEI Company), operated at 200 keV.

#### 2.7.1 BLAST

NCBI nucleotide BLAST 2.6.1 (https://blast.ncbi.nlm.nih.gov/Blast.cgi) was used with NT4 as the query sequence and mouse genomic + transcript was chosen as the database.

#### 2.7.2 NT4 qPCR in mouse fibroblasts

To assess the possibility of detecting NT4 from human CDC‐sEVs in mice, RNA was isolated from mouse (Cell Biologics C57‐6067) and human (ATCC PCS‐201‐012) dermal fibroblasts at passage 4 using miRNeasy Mini Kit (Qiagen). miScript II RT Kit (Qiagen) was used for reverse transcription and qPCR was performed using QuantiTect SYBR Green (Qiagen) on a QuantStudio 12K Flex system (Applied Biosystems), with QuantiMir universal reverse primer, NT4 forward primer (5′–GGTCCGATGGTAGTGGGTTATCAG–3′), and mouse U6 forward primer (5′–TGGCCCCTGCGCAAGGATG–3′) for the housekeeping gene. Fold change was calculated using 2^(‐ΔΔCt)^, where ΔCq compares the NT4 Cq value to the U6 Cq value of the same sample, and ΔΔCq compares ΔCq to that of mouse fibroblasts, normalizing the fold change of mouse fibroblasts to 1.

#### 2.7.3 QPCR Efficiency

Serial dilutions of CDC‐sEVs or IMEX were added to homogenized liver tissue from a healthy mouse. After RNA isolation using the miRNeasy Mini Kit (Qiagen), reverse transcription was performed using the miScript II RT Kit (Qiagen). qPCR was done using QuantiTect SYBR Green (Qiagen) on QuantStudio 12K Flex instrument (Applied Biosystems) with the QuantiMir universal reverse primer, NT4 forward primer, and mouse U6 forward primer the housekeeping gene. ΔCq was determined by subtracting the Cq value for U6 from that of NT4 of the same sample. ΔCq was plotted versus the log of the number of CDC‐sEVs in the sample. Linear regression analysis was performed using GraphPad Prism 7. Efficiency was calculated for all the other organs (heart, kidney, spleen, brain, lungs) using CDC‐sEVs (derived from the same batch).

#### 2.7.4 QPCR Specificity

Dissociation curves were prepared from the SYBR Green qPCR assay using a temperature gradient from 60°C to 95°C. The negative first derivative of the fluorescence curve was taken, and data from technical triplicates were graphed as fluorescence derivative versus temperature.

#### 2.7.5 Femoral vein injection in healthy animals

In vivo, experimental protocols were performed on 10‐ to 12‐week‐old male C57BL76J mice (Jackson Laboratory). Mice were housed under pathogen‐free conditions in a temperature‐controlled room with a 12‐h photoperiod. CDC‐sEVs (2 × 10^9^/mouse, corresponding to 141.15 ± 7.4 μg) or IMEX (2 × 10^9^/mouse, corresponding to 85.71 ± 4.05 μg) were resuspended in 100 μl of serum‐free IMDM and injected into the femoral vein of healthy mice anaesthetized by isofluorane. Vehicle‐treated mice received an equal volume of serum‐free IMDM injected into the femoral vein. After 1 h, mice were sacrificed and tissues (heart, liver, kidney, spleen, brain, lungs) were removed (without prior perfusion), washed multiple times, minced, weighed (20 mg/organ), and stored in Qiazol at ‐80°C until further processing was performed. Blood was collected from the hepatic vein (using K2 EDTA tubes), centrifuged 3000 g 15 min at 4°C and plasma was isolated fresh for EV purification and RNA isolation. Urine was collected from the bladder of animals and EV‐RNA was isolated using a urine EV RNA isolation kit (Norgen Biotek, cat# 47200).

#### 2.7.6 Retro‐orbital injection in healthy animals

For retro‐orbital injections, mice were anaesthetized by isofluorane and CDC‐sEVs (100 ul in IMDM) or vehicle (IMDM only) injected in the retro‐bulbar space. Mice were sacrificed after 1 h, and tissues (heart, liver, kidney, spleen, brain, lungs), plasma, and urine were collected without prior perfusion.

#### 2.7.7 Femoral vein injection in animals with ischemia/reperfusion injury

To induce ischemia/reperfusion (I/R) injury, mice were provided general anaesthesia, and then a thoracotomy was performed at the 4th intercostal space to expose the heart and left anterior descending coronary artery. A 7–0 silk suture was then used to ligate the left anterior descending coronary artery, which was subsequently removed after 45 min to allow for reperfusion for 20 min. Vehicle (IMDM only) or CDC‐sEVs (2 × 10^9^ particles in 100 μl IMDM) were injected into test animals via slow femoral vein injection. After 1 h the animals were sacrificed and whole organ tissues, plasma, and urine were collected without prior perfusion. For inhibition studies, 200 units of Heparin (1000 USP units/ml, Sagent Pharmaceuticals) or 30 mg/kg of Dynasore hydrate were injected intraperitoneal (IP) in a total volume of 200 μl 10 and 15 min, respectively, before EVs injection.

#### 2.7.8 TTC staining

Two days following the I/R injury, 10% KCL was injected into the LV to arrest hearts in diastole. Then, hearts were harvested, washed in PBS, and then cut into 1‐mm sections from apex to base, above the infarct zone. Sections were incubated with 1% solution 2,3,5‐triphenyl‐2H‐tetrazolium chloride (TTC) for 30 min at 37C in the dark and washed with PBS. Then, sections were imaged and weighed. The infarcted zones (white) were delineated from viable tissue (red) and analyzed (ImageJ software). Infarct mass was calculated in the tissue sections according to the following formula: (infarct area/tot area) × weight (mg). For TTC staining 100 units of Heparin (1000 USP units/ml, Sagent Pharmaceuticals) were used due to the high mortality of the protocol.

#### 2.7.9 QPCR

Tissues were homogenized in Qiazol (Qiagen) using Bead Ruptor 12 (OMNI International) with RNase‐free steel beads. RNA was isolated using the miRNeasy Mini kit (Qiagen). Exosomal RNA from the plasma and the urine was isolated using ExoQuick Exosome Isolation and RNA Purification Kit (for Serum & Plasma) (System Biosciences) and Urine Exosome Isolation kit (Norgen), respectively, according to the manufacturer's protocol. RNA was resuspended in 15 μl RNase free water and RNA concentration and purity were determined using a NanoDrop Spectrophotometer (Thermo Scientific). miScript II RT Kit (Qiagen) was used for reverse transcription using 2 μg of isolated RNA for organs, 100 ng RNA for plasma, and 60 ng for urine, and 5× miScript HiFlex Buffer. cDNA was diluted 1:10 for organs or used undiluted for plasma and urine and used immediately for qPCR. qPCR was done with QuantiTect SYBR Green (Qiagen) on QuantStudio 12K Flex or QuantStudio 6 Flex system (Applied Biosystems) with the QuantiMir universal reverse primer, NT4 forward primer (10 μM), and mouse U6 forward primer (10 μM) for the housekeeping gene. qPCR was run using the following protocol: initial activation step 15 min 95°C, 3‐step cycling (Denaturation 15 s 94°C, Annealing 30 s 60°C, Extension 30 s 70°C) for 45 cycles. ΔCq was determined by subtracting the Cq value for U6 from that of NT4 of the same sample. The number of CDC‐sEVs in the sample was calculated using the formula obtained from the standard curve of efficiency, obtaining the number of EVs found in all the organs analyzed and expressed as Log(EVs)/mg and Log(EVs)/organ. Percentage values were calculated based on the injected EVs dose (2 × 10^9^) EVs/animal. To determine plasma concentration we used a total plasma volume of 350 μl (from 700 μl of blood; graphical data adjusted to EVs/100 μl).

### 2.8 Next‐generation extracellular vesicle RNA sequencing

Total EV RNA was isolated from serum‐free conditioned media of CDCs EVs and normal human dermal fibroblast EVs using a Urine EV RNA Isolation Kit (Norgen Biotek). Total RNA sequencing was done using System Biosciences Inc's ExoNGS service. Briefly, samples were sequenced using the Illumina platform's NextSeq High Output single‐end sequencing with NextSeq 500/550 High Output v2 kit (Illumina) per technical instructions. Data processing was done using a cloud‐based RNA seq analysis kit (Maverix, Analytic Platform, System Biosciences Inc). Reads were curated for quality including filtering out including adaptor dimer sequences. The remaining reads were enumerated, and sequences were aligned to reference genome (human) using Bowtie (Langmead et al., 2009) and only mappable reads were considered for evaluation.

### 2.9 Cell isolation and in vitro uptake experiments

#### 2.9.1 Rat Bone marrow‐derived macrophage preparation

Femurs were isolated from 7‐ to 10‐week‐old Wistar–Kyoto rats. Bone marrow was isolated by flushing with PBS then filtering through a 70‐μm mesh. Red blood cells were lysed with ACK buffer (Invitrogen) and then resuspended in IMDM (Gibco) containing 20 ng/ml M‐CSF (eBioscience) for plating. The media was exchanged every 2–3 days until day 5, at which point bone marrow‐derived macrophages (BMDMs) were used for in vitro experiments.

#### 2.9.2 Neonatal rat ventricular myocyte isolation

Neonatal rat ventricular myocytes (NRVMs) were isolated from P2 neonatal Sprague–Dawley rats as previously described (Sekar et al., 2009). The cells were plated on fibronectin‐coated 12‐well plates at a density of 0.5 million cells/well in Dulbecco's Modified Eagle Medium (DMEM) containing 10% Fetal Bovine Serum (Gibco) media, and incubated at 37°C, with 5% CO2 for 24 h.

#### 2.9.3 Rat cardiac fibroblast isolation

Cardiac fibroblasts were isolated from the first and second pre‐plating from NRVM isolation procedure and cultured in DMEM containing 10% Fetal Bovine Serum (Gibco), and incubated at 37°C, with 5% CO_2_ until confluence and used at p2 and p3.

#### 2.9.4 Rat cardiac microvascular endothelial cells

Rat endothelial cells were purchased from Cell Biologics (RN‐6024) and cultured in rat endothelial cell medium (Cell Biologics) containing 2% FBS.

#### 2.9.5 In vitro uptake and uptake inhibition

For in vitro uptake experiments cells were treated in a 1:100 ratio of cells to EVs in a medium with 2% serum. After the desired incubation period, cells were washed with phosphate‐buffered saline (PBS) two times, then Qiazol was added directly into the 12‐well plate. RNA was isolated using a miRNeasy Mini Kit (Qiagen) according to the manufacturer's protocol. miScript II RT Kit (Qiagen) was used for reverse transcription using 100 ng of isolated RNA 5x miScript HiFlex Buffer. cDNA was used undiluted for qPCR. qPCR was done with QuantiTect SYBR Green (Qiagen) on QuantStudio 12K Flex or QuantStudio 6 Flex system (Applied Biosystems) with the QuantiMir universal reverse primer, NT4 forward primer(10uM), and rat RNU1A forward primer(10uM) for the housekeeping gene. For uptake inhibition, experiments rat cardiac fibroblast were pre‐treated with or without the inhibitors heparin (10 and 50 μg/ml) and dynasore (50 and 100 μM) for 30 min. Cells were then incubated with CDC‐sEVs (1:100) for 2 h, washed with PBS two times, then Qiazol was added directly into the 12‐well plate followed by RNA extraction and qPCR for NT4 expression levels.

### 2.10 Statistical analysis

Statistical comparisons between groups of two were made using an independent one‐tailed or two‐tailed independent Student's *t*‐test with a 95% confidence interval. Comparisons made between groups of three or more were made using a one‐way analysis of variance with Tukey's post‐test to control for multiple comparisons.

## 3 RESULTS

### 3.1 NT4 is absent in mouse tissue and highly enriched in human CDC‐sEVs

**FIGURE 1:**
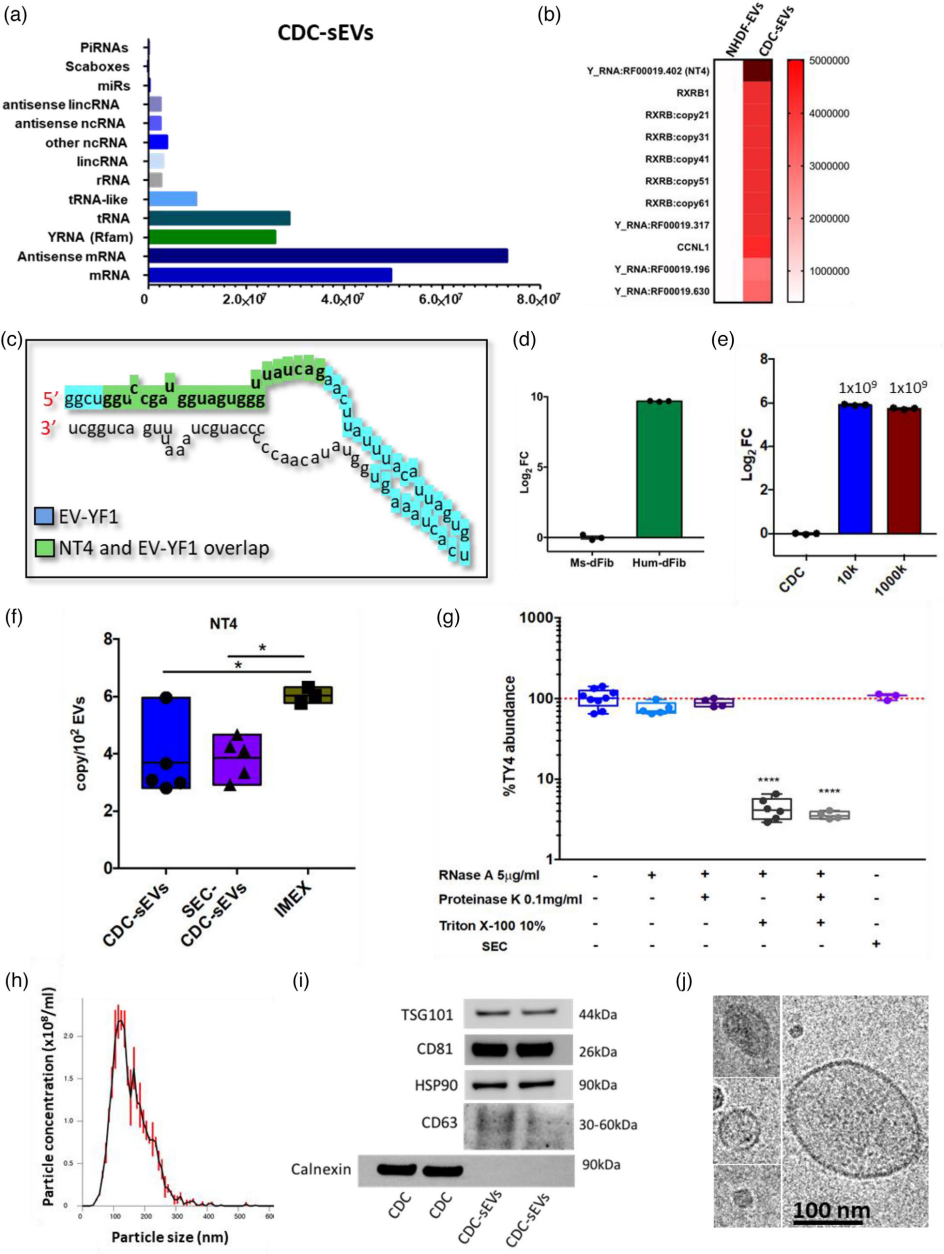
Native hY4_f_ Tracer (NT4) is absent in mouse tissue and highly enriched in human CDC‐sEVs. (a) Next‐generation sequencing of human CDC‐sEVs identified a diversity of RNA species (PIWI RNA (piRNA), microRNA (miR), long intervening non‐coding RNAs (lincRNA), non‐coding RNAs (ncRNA), ribosomal RNA (rRNA), transfer RNA (tRNA), messenger RNA (mRNA). (b) Sequencing of human CDC‐sEVs identified a fragment of YRNA 4 (dubbed NT4) as the single most abundant RNA constituent compared to NHDF‐EVs (normal human dermal fibroblast EVs; NHDF‐EVs). (c) NT4 (highlighted in green) is a smaller portion of a 57‐nucleotide fragment of the YRNA 4 gene (EV‐YF1; highlighted in blue) previously identified by our group. (d) NT4 abundance by qPCR in mouse dermal fibroblasts (Ms‐dFib) and human dermal fibroblasts (Hum‐dFib) expressed in Log_2_ fold change (*n *= 3 biological replicates per group; data presented as mean ± SEM). (e) qPCR for NT4 abundance among different EV preparations isolated from less pure (10 kDa(10K)) and purer (1000 kDa(1000K) ultrafiltration columns) (*n *= 3 biological replicates per group; data presented as mean ± SEM). (f) NT4 abundance by qPCR in CDC‐sEVs isolated using 10 kDa immortalized ultrafiltration columns (CDC_EVs), CDC‐EVs isolated using size‐exclusion chromatography (SEC) and immortalized CDC‐derived EVs (IMEX); (*n *= 3–5 replicates per group, comparisons (g), and were analyzed using one‐way ANOVA with Tukey's post‐test * = *p* < 0.05). (g) NT4 RNA is contained inside CDC‐sEVs as shown by protection from RNase degradation and proteinase K treatment and SEC isolated sEVs (*n *= 4–8 replicates per group, mean ± SEM, significance was determined using one‐way ANOVA with Tukey's post‐test **** = *p* < 0.0001). (h) Nanoparticle tracking analysis revealing particle size distribution and concentration of CDC‐sEVs. (i) Western blot for typical EV markers (TSG101, CD81, HSP90, CD63). CDC‐sEVs are also negative for Calnexin. (j) CDC‐sEVs isolated visualized by transmission electron microscopy (TEM)

We sought to develop a method of detecting native (unmodified) CDC‐sEVs in vivo by exploiting the sensitivity of quantitative PCR to detect species‐specific EV‐enriched RNA species in host tissue. MicroRNAs (miRs) are among the most highly studied RNA species of the EV payload, but their remarkable evolutionary conservation across species severely limits their utility as unique tracer molecules. Next‐generation sequencing of human CDC‐sEVs identified a diversity of RNA species (Figure 1a). We identified a 24‐nucleotide 5′ fragment of human Y RNA4 we dub native tracer 4 (NT4). NT4 is the single most abundant RNA species in human CDC‐sEVs (Figure 1b) and is enriched in CDC‐sEVs compared to a therapeutically inert EV used frequently by our group as a comparator (normal human dermal fibroblast EVs; NHDF‐EVs). NT4 spans a portion of a 57‐nucleotide Y RNA4 fragment (EV‐YF1) we have identified previously (Figure 1c) (Cambier et al., 2017). *In silico* analysis using an RNA structure prediction algorithm suggests that NT4 may assume two energetically probable stem‐loop secondary structures characteristic of Y RNA molecules (Figure S1a) (Gruber et al., 2008). To evaluate the specificity of NT4, we performed an NCBI BLAST search (Altschul, 1997) to probe for homologs of NT4 in the mouse transcriptome. No identical matches were identified; the closest match shared 83% sequence identity (20/24 nt) and mapped to mouse RNA Y1 (mice lack Y RNA4; Figure S1b). Therefore, we designed specific primers for NT4 amplification that completely cover the NT4 sequence and excludes areas of high sequence identity with mouse RNA Y1 (Figure 1Sb).

To test the specificity of our primers, we performed qPCR for NT4 in mouse fibroblasts compared to human fibroblasts. Human fibroblasts were more than one‐thousand‐fold enriched in NT4 compared to mouse fibroblasts as measured by qPCR (Figure 1d). These results validate the specificity of our primer set and exclusivity of NT4 in human tissue compared to murine tissue. To quantify NT4 in CDC‐EV preparations, we generated a standard curve using known copies of synthetic NT4 RNA (Figure 1c). EV preparations isolated from 10 and 1000 kDa ultrafiltration columns had comparable abundance of NT4 confirming that NT4 is EV‐derived and not a co‐purified contaminant (Figure 1e). This was further confirmed by analysis of NT4 using SEC (Figure 1f). We further evaluated whether NT4 can also be used to track EVs from immortalized CDCs (IMEX). In IMEX, the abundance of NT4 was significantly higher than CDC‐EVs; Figure 1f). This is higher than reported copy numbers of small RNAs, namely miRs. For most miRs investigated, the reported abundance (copies per EV particle) for comparable EV isolation methods range from 1:100 to 1:10,000 (Chevillet et al., 2014). We confirmed that NT4 is contained within EVs using an RNAse and proteinase protection assay and EVs purified by SEC (Figure 1g). Size distribution and EV protein expression were measured using Nanosight© tracking analysis (NTA; Figure 1h), western blot for typical EV markers (Figure 1i), and protein array respectively (Figure 1e), according to MISEV2018 guidelines (Thery et al., 2018). To assess the purity of CDC‐sEV preparations, particles per microgram of protein were measured (10^7^ particles per microgram of protein) which reflected the abundance of protein using this isolation method (Figure S1d). Transmission electron microscopy (TEM) was made to confirm the structure of pCDC‐sEVs (Figure 1j).

### 3.2 NT4 amplification is efficient, specific, and sensitive in mouse tissue

**FIGURE 2:**
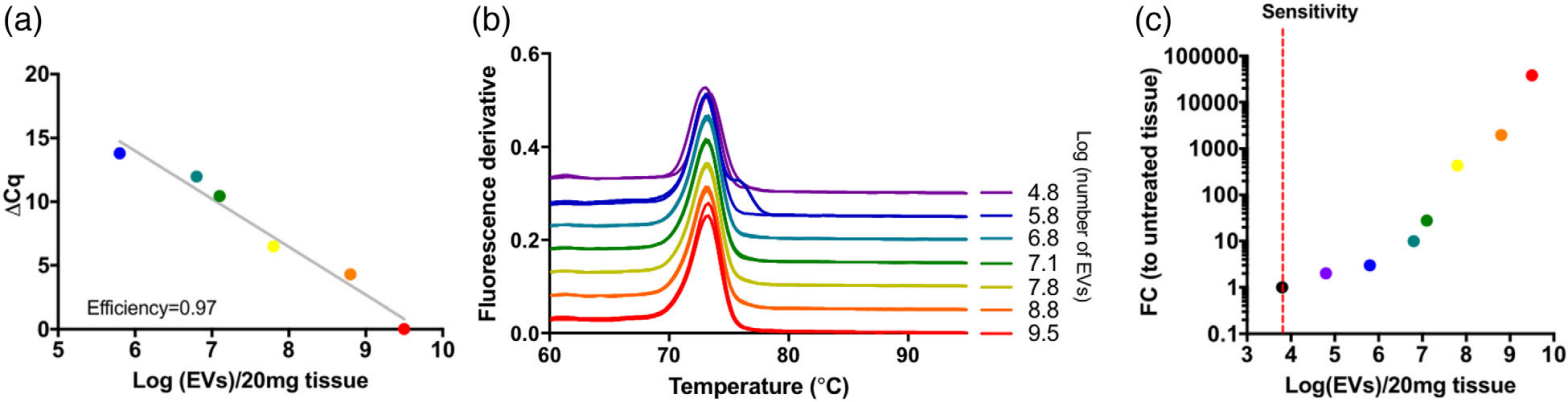
NT4 amplification is efficient, specific, and sensitive in mouse tissue. (a) Efficiency of NT4 amplification using a standard curve prepared using serial dilutions of known numbers of CDC‐sEVs spiked into 20 mg samples of homogenized mouse liver tissue (ΔCq is calculated by subtracting the U6 Cq value to the NT4 Cq value of the same sample). (b) Specificity of the NT4 amplification demonstrated by SYBR green dissociation curves (see Methods). Curves are offset by better visibility. A single dissociation peak near 73°C indicates specificity, whereas multiple peaks or different melting temperatures indicate non‐specific amplification. (c) The same data from (a) were graphed as fold change ([FC] to untreated tissue) versus EVs found in 20 mg of liver tissue. The limit of reliable detection sensitivity is 10^4^ EVs/20 mg tissue. The colour of each data point is consistent across (a–c)

To assess the efficiency, sensitivity, and specificity of NT4 qPCR amplification in animal tissue, we generated a copy number curve using known numbers of CDC‐sEVs spiked in mouse liver tissue. The background quantification cycle (Cq) value of NT4 in naïve mouse tissue is low with an average cycle value of 38. The NT4 qPCR has an efficiency of 0.97 (Figure 2a), the SYBR Green dissociation curve shows a distinct single peak at the melting temperature in relevant concentrations (Figure 2b), indicating the specificity of the primers with a detection limit of 10^4^ per 20 mg of tissue (Figure 2c).

To further optimize the sensitivity and specificity of detection, we optimized different variables that can impact qPCR assays, including annealing temperature, amount of input RNA for reverse transcription, and cDNA for amplification. Increasing annealing temperature (from 55 to 60°C) we can decrease the background while maintaining the specificity of NT4 detection. We found 2 μg to be the best RNA concentration to be used for RT reaction, and a subsequent dilution of cDNA of 1:10 for qPCR (Figure S2a and b). An annealing temperature of 60°C was used for all the experiments in the manuscript. A copy number curve using known numbers of CDC‐sEVs spiked in mouse tissues was performed for all the organs (heart, kidney, spleen, brain, lungs) (Figure S2f‐j).

### 3.3 Biodistribution of intravenously infused CDC‐sEVs in healthy animals

**FIGURE 3:**
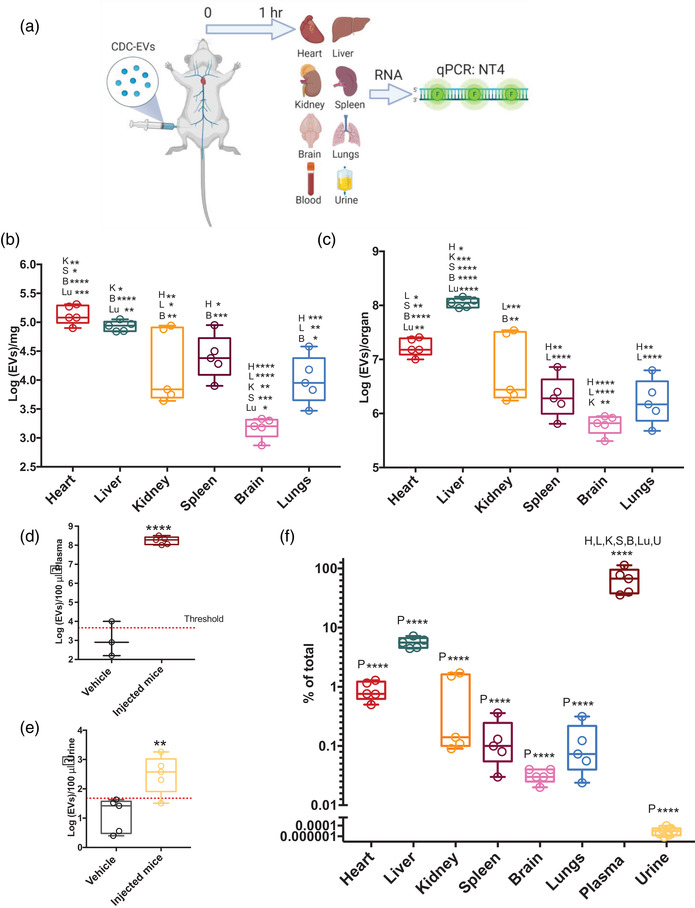
CDC‐sEVs biodistribution in healthy animals after femoral vein injection. (a) Schematic of the experimental design. Healthy mice received an intravenous (IV) injection of 2 × 10^9^ CDC‐sEVs or vehicle control (serum‐free media) into the femoral vein. Heart, liver, kidneys, spleen, brain, lungs, plasma, and urine were collected. (b) Biodistribution data in tissue 1 h after administration of CDC‐EV expressed in Log (EVs)/mg of tissue, whole organ (c), plasma (d), and urine. (f) Percentage of total injected EVs detected in different organs. All data is presented as mean ± SEM (*n* = 5 animals per group), comparisons in (a–f) were analyzed using one‐way ANOVA with Tukey's post‐test * = *p* < 0.05, ** = *p* < 0.01, and *** = *p* < 0.001 **** = *p* < 0.0001). Abbreviations above box plots reflect statistic differences between sample and other organ tissue (B, brain; Lu, lung; H, heart; K, kidney; S, spleen; L, liver; P, Plasma; U, urine)

Having identified an EV‐associated tracer and validated a reliable strategy for tracking unmodified EVs, we sought to understand how CDC‐sEVs distribute under healthy and diseased conditions. Because CDC‐sEVs are used primarily for heart disease indications we compared CDC‐sEV biodistribution in healthy animals and animals with cardiac ischemia/reperfusion (I/R) injury. For translational relevance, we used intravenous delivery as our route of choice for administration. Based on preliminary data we determined that 1 h is the optimal time point for the highest sensitivity. To determine the biodistribution of CDC‐sEV in healthy animals, 10‐12‐week‐old female mice were injected intravenously via the femoral vein with 100 μl of serum‐free media containing 2 × 10^9^ EVs or serum‐free media only (vehicle). One hour after administration mice were sacrificed and RNA from primary target organs and body fluids including the heart, liver, kidneys, spleen, brain, lungs, plasma, and urine were isolated for quantification of NT4 copies (Figure 3a). Tissue was washed multiple times to wash away the blood that might confound results.

Using a standard curve of animal tissue containing known numbers of CDC‐sEVs, we calculated the number of EVs present per mg of tissue or microliter of plasma or urine (Figure S2c and e). In agreement with previous reports (Choi & Lee, 2016; Di Rocco et al., 2016), CDC‐sEVs distributed primarily to the liver (Hwang et al., 2015; Morishita et al., 2015). In contrast with previous findings, however, the second most retaining was the heart followed by the kidneys (Figure 3b). Per mg, cardiac tissue retained more EVs than other organs (Figure 3c). Furthermore, at 1 h post‐administration, a significant portion of the EVs remained in circulation (Figure 3d). EVs were also detected in urine, indicating rapid clearance (Figure 3e). Compared to the original administered amount DUST could account for approximately 74% of injected EV dose (Figure 3f). It is likely the remainder of the particles were fixed by complement and subsequently taken up and destroyed by macrophages and other phagocytes (Imai et al., 2015; Jeon et al., 2017; Wassef et al., 1991).

Animals that were perfused prior to organs collection show a similar biodistribution profile (Figure S3a and b). The biodistribution of CDC‐sEVs purified using SEC method was also assessed in organs and plasma (Figure S3c–e). To determine whether engineering of the producer cell (i.e., immortalization) affected the distribution of derived EVs, we evaluated the biodistribution of EVs from IMEX (Figure S3a). As scale‐up efforts turn to therapeutic cell immortalization investigating the effects on biodistribution becomes a critical aspect of translation to consider. Similar to CDC‐sEVs, the liver and heart were the primary locations for EVs biodistribution, followed by the kidney (Figures S3b–d). In conclusion, sEVs from immortalized CDCs had a similar biodistribution profile compared to those derived from primary CDCs.

To evaluate whether the site of intravenous administration affects biodistribution we used retro‐orbital venous sinus administration as a comparator to injection through the femoral vein (Figure S4a). Like femoral vein injection, retro‐orbital administration concentrated EVs into the liver, followed by the heart and kidney. Significantly higher levels of EVs in the brain were detected with retro‐orbital administration and lower levels in the lungs (Figures S4b, c, and g). Also similar to femoral vein injection, a majority of the administered EVs remained in circulation at 1 h post‐administration (Figure S4d) and were detectable in the urine (Figure S4e). Taken together, retro‐orbital administration of EVs results in similar biodistribution except for elevated retention in brain tissue and lower retention into the lungs (Figure S4g).

### 3.4 Biodistribution of intravenously infused CDC‐sEVs in animals with cardiac injury

**FIGURE 4:**
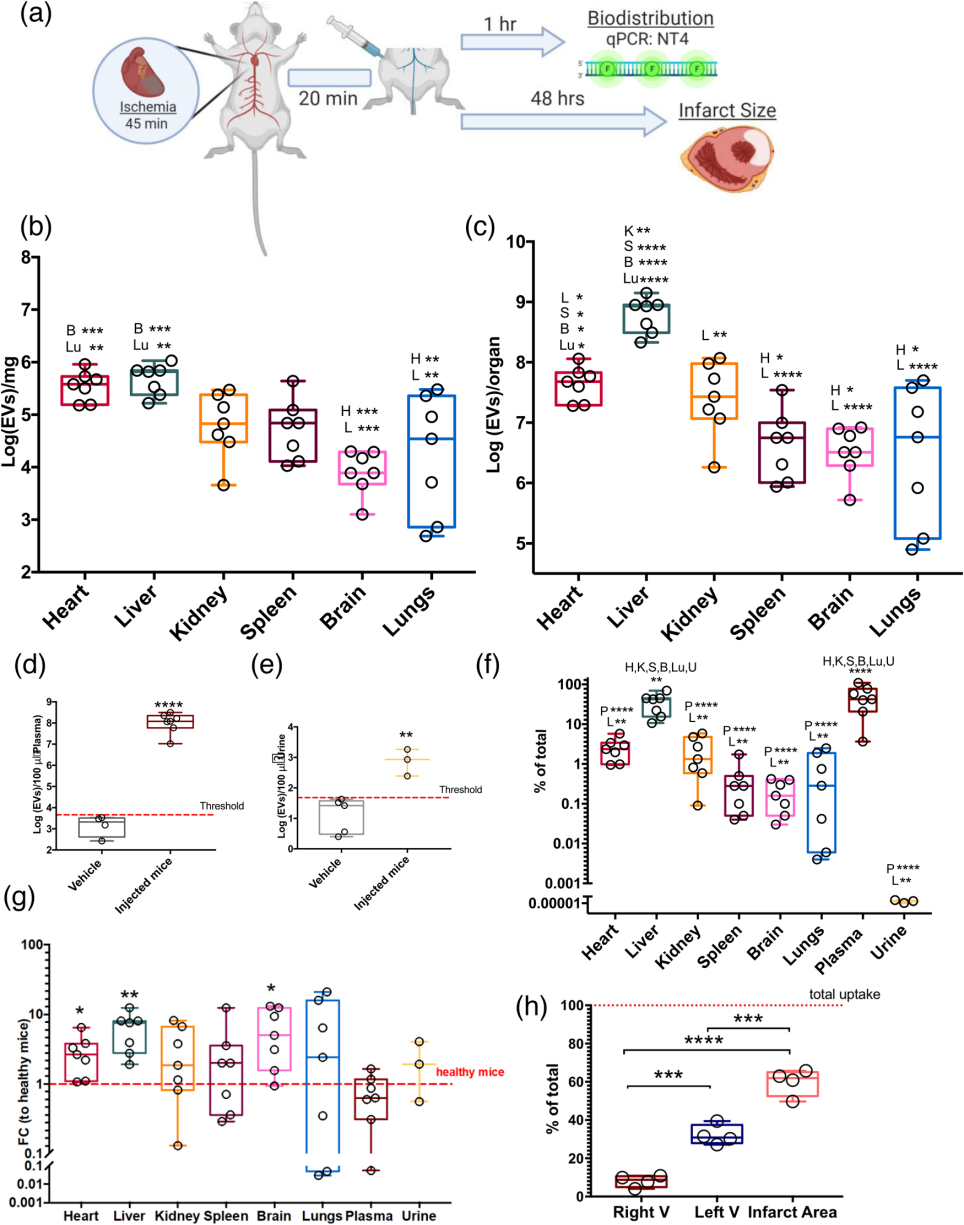
CDC‐sEVs distribute differently between healthy and injured animals. (a) Schematic of the experimental design. Mice underwent a protocol of ischemia/reperfusion (45 min of ischemia followed by reperfusion) followed by an intravenous (IV) injection of 2 × 10^9^ CDC‐sEVs or vehicle control (serum‐free media) into the femoral vein 20 min after the reperfusion. Heart, liver, kidneys, spleen, brain, lungs, plasma, and urine were collected. (b) CDC‐EV distribution per mg of tissue (b), whole organ (c), plasma (d), and urine (e) 1‐h post‐administration (*n* = 7 animals per group except (d and e): *n* = 3–6 animals per group). (f) Percentage of total injected EVs detected in different organs (g) Difference in percentage distribution in animals with ischemia/reperfusion injury (I/R) compared to healthy animals. (h) Percentage of injected EVs detected in different areas or cardiac tissue 1 h after IV administration (*n* = 4 animals per group). All data presented as mean ± SEM. Comparisons in (a–c), (g), and (h) were analyzed using one‐way ANOVA with Tukey's post‐test * = *p* < 0.05, ** = *p* < 0.01 *** = *p* < 0.001 and **** = *p* < 0.0001). Comparisons in (d, e, and g) were analyzed using *Student's independent t‐test* * = *p* < 0.05, ** = *p* < 0.01 and **** = *p* < 0.0001). Abbreviations above box plots reflect statistic differences between sample and other organ tissue (B, brain; Lu, lung; H, heart; K, kidney; S, spleen; L, liver; P, Plasma; U, urine)

We have shown in previous work (using a fluorescently labelled strategy) that CDC‐sEVs target the infarcted myocardium when delivered via the intracoronary route 20 min following reperfusion (De Couto et al., 2015). We sought to assess the therapeutic capacity and biodistribution of CDC‐sEVs in the mouse I/R model. Injured animals received femoral vein infusion of CDC‐sEVs 20 min after reperfusion injury (Figure 4a). Animals underwent transient coronary ligation to induce I/R. Major organs (heart, liver, kidneys, spleen, brain, and lungs) were harvested 1 h after EV injections. As observed in the healthy model, the liver, heart, and kidneys were major locations of EV biodistribution (Figures 4b and c), with the majority of EVs retained in circulation (Figure 4d). EVs were also detected in the urine (Figure 4e). In the I/R model, we were able to account for about 93% of injected EVs (Figure 4f), significantly higher than in healthy protocol. Animals with I/R injury retained higher levels of CDC‐sEVs in the heart, liver, and brain compared to healthy hearts with similar trends observed in other organs (though did not reach the level of statistical significance; Figure 4g). These findings support early observations showing that modification of the surface of injured cells facilitates EV trafficking and uptake, especially in injured muscle tissue (Bittel & Jaiswal, 2019). To determine whether the site of injury retained higher amounts of EVs, we obtained tissue from the right and left ventricles, and the infarcted region. In the ischemia‐reperfusion model of cardiac injury, the left anterior descending coronary artery which perfuses 50%–60% of the left ventricle is temporarily occluded minutes followed by reperfusion. This leads to ischemic injury and damage to the left ventricle with no direct insult to the right ventricle. In congruence with previous findings by us and others, EVs were retained in the infarct area more than un‐infarcted left ventricular tissue or the right ventricle (Figure 4h).

**FIGURE 5:**
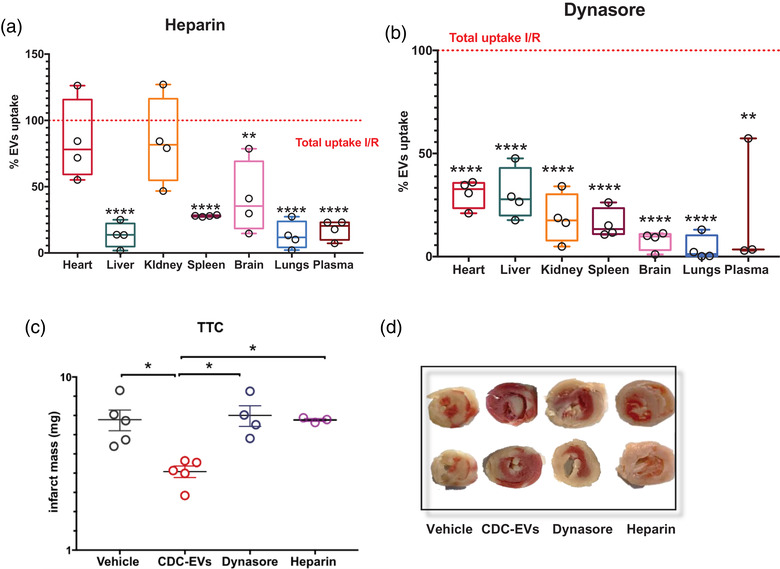
Inhibition of EVs uptake led to abrogation of therapeutic effect in I/R mouse model. I/R injured animals treated with CDC EVs with co‐treatment of heparin (a) or dynasore (b) compared to CDC‐EV treatment only. Data are expressed as the percentage of injected EVs detected in each organ compared to those in I/R animals treated with a single injection of CDC‐sEVs only (*n* = 4 animals per group. In dynasore‐treated animals one value for EV uptake in the blood was excluded). All data presented as mean ± SEM. Comparisons in (a and b) were analyzed using *Student's independent t‐test* ** = *p* < 0.01 and **** = *p* < 0.0001). (c) Quantification of infarct area in each group and representative images of TTC stained heart sections (d). Infarct mass (white area) was calculated in the tissue sections according to the following formula: (infarct area/tot area) × weight (mg) (*n* = 3, 4, or 5 animals/group, mean ± SEM, significance was determined using one‐way analysis of variance with Tukey's post‐test with * = *p* < 0.05)

To dissect the mechanism of EV uptake in tissue, we blocked EV surface docking, clathrin‐mediated endocytosis, and macropinocytosis using heparin and dynasore, respectively. Intraperitoneal administration of heparin led to significant reductions in uptake in several organs including the liver, spleen, brain, and lungs. Surprisingly, it also decreased the retention of EVs in circulation which may be related to heparin‐mediated macrophage mobilization (Bruno et al., 2018) (Figure 5a). Unexpectedly, retention by cardiac tissue was unaltered which may suggest heparan‐sulfate proteoglycan (HSPG)‐independent EV docking mechanism in cardiac tissue (Figure 5a). Animals treated with dynasore, however, had dramatic reductions of EV retention in all tissue (Figure 5b). This demonstrates that retention of EVs in tissue is not driven by accumulation in capillary beds (in the extracellular space) but rather on clathrin‐dependent cellular uptake and macropinocytosis of EV particles. Surprisingly, we also observed significantly lower retention of EVs in plasma following dynasore and heparin treatment (Champanhac et al., 2021). Heparin and dynasore prevent the uptake of EVs through heparin sulfate proteoglycan‐dependent mechanisms (Christianson et al., 2013) which includes phagocytosis (Fukasawa et al., 1997; Marie‐Anaïs et al., 2016). The reticuloendothelial system functions to clear material from circulation through either saturable (phagocytosis by leukocytes in the liver and lymphoid tissue). or non‐saturable means (catabolism and renal excretion) (Boneu et al., 1990). Saturation by heparin or disruption by dynasore of the former mechanism leaves renal and hepatobiliary clearance as the likely route of clearance for EVs in heparin‐treated or dynasore‐treated animals. Given the inhibitory effect of heparin and dynasore on EV uptake by macrophages, the CDC‐sEVs effect is impaired.

To investigate whether inhibition of uptake led to abrogation of therapeutic effect, hearts were excised and stained with TTC to identify scar size 48 h after EVs administration (Figure 5b and c). Animals infused with CDC‐sEVs had reduced infarct sizes compared to controls while animals receiving CDC‐sEVs with heparin or dynasore had scar sizes similar to vehicle controls. Interestingly, despite retention of uptake capacity by the heart with heparin treatment, the CDC‐EV effect was attenuated. In previous work by our group, we showed that macrophages play an integral role in the mechanism of action of CDCs and their EVs (de Couto et al, 2015, 2017). Therefore, inhibition of EV docking or endocytosis abrogated their cardioprotective effect.

### 3.5 CDC‐sEVs are uptaken more efficiently by macrophages, endothelial cells, and cardiac fibroblasts compared to cardiomyocytes

**FIGURE 6:**
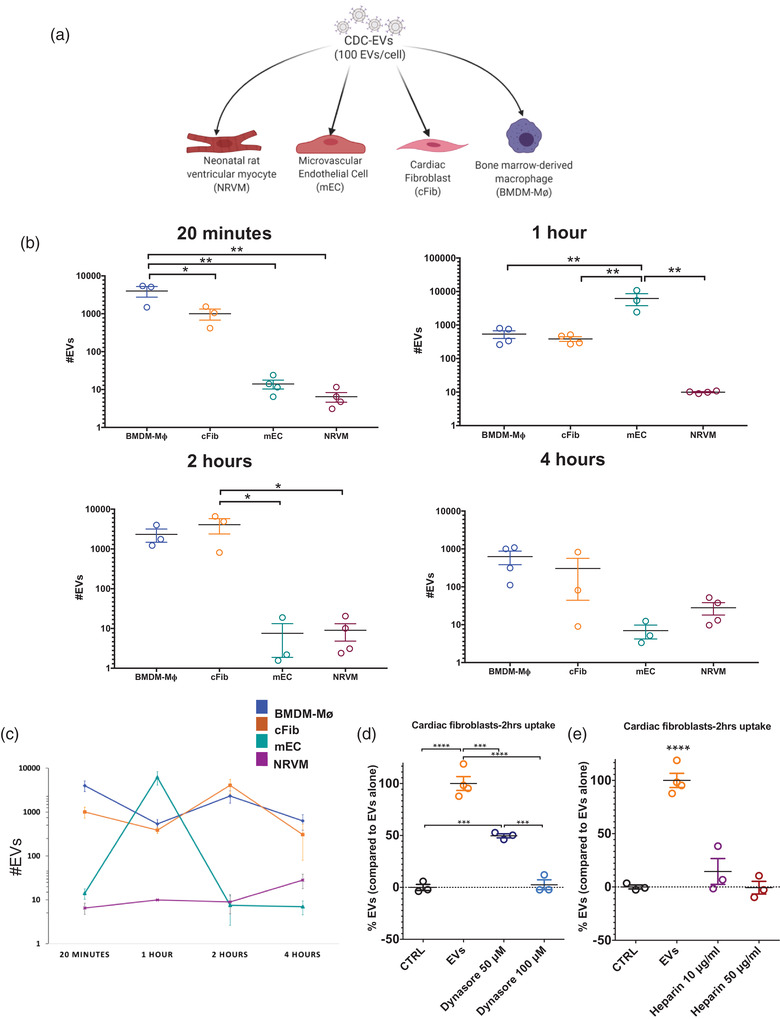
CDC‐sEVs are taken up more efficiently by macrophages, cardiac fibroblasts, and endothelial cells compared to cardiomyocytes. (a) Schematic of the experimental design. Neonatal rat ventricular myocytes (NRVM), Microvascular Endothelial cells (mEC), Cardiac Fibroblasts (cFib), and bone marrow‐derived macrophages (BMDM‐Mø) were used in the experiment. (b) Number of EVs detected in the four cell types at various time points post‐EV treatment (20 min, 1, 2, 4 h) (*n *= 4 biological replicates per group; data presented as mean ± SEM). (c) Data from in vitro uptake for the different cell types plotted together (*n *= 4 biological replicates per group; data presented as mean ± SEM). (d and e) Number of EVs (expressed as a percentage) detected in cardiac fibroblasts pre‐treated with or without the uptake inhibitors dynasore (d) (50 μM, 100 μM) and heparin (e) (10 and 50 μg/ml) for 30 min. Cells were then incubated with CDC‐sEVs (1:100) for 2 h with CDC‐sEVs (*n* = 3–4 biological replicates per group; data presented as mean ± SEM, significance was determined using one‐way analysis of variance with Tukey's post‐test with * = *p* < 0.05, ** = *p* < 0.01, *** = *p* < 0.001, and **** = *p* < 0.0001)

Finally, to more finely dissect the uptake kinetics of individual cell types that make up the cardiac microenvironment, we evaluated the rate and amount of CDC‐EV uptake in NRVM, cardiac fibroblasts, microvascular endothelial cells and bone marrow‐derived macrophages (Figure 6a) in vitro. Uptake was measured at 20 min, 1, 2 h, and 4 h after exposure to CDC‐sEVs. Variable kinetics of CDC‐EV uptake were observed in between the cells tested (Figure 6b). Expectedly, macrophages had the fastest and highest overall uptake, followed by endothelial cells, and cardiac fibroblasts. NRVMs had the slowest and lowest overall uptake of EVs (Figure 6c). These results suggest that macrophages, cardiac fibroblasts, and endothelial cells are the effective recipients of CDC‐sEVs and that the direct effect of CDC‐sEVs on myocytes is likely nominal (Figure 6d). Uptake was inhibited in cardiac fibroblasts with the use of the inhibitors heparin and dynasore (Figure 6d and e). Moreover, once NT4 is taken up, the signal localizes mostly to the cytosol compared to the nuclear fraction (Figure S6a).

## 4 DISCUSSION

EVs represent attractive next‐generation therapeutics. As cell‐free agents, they circumvent key challenges of cell therapy and are a highly adaptable platform (Wiklander et al., 2019). Despite intense research in the EV field, relatively few studies have been dedicated to EV biodistribution; a majority of the reports have been accessory to EV translational research objectives. The current state‐of‐the‐art relies primarily on the optic resolution to detect particles in tissue including staining with lipophilic fluorescent dyes (El Andaloussi et al., 2013; Grange et al., 2014; Ohno et al., 2013; Sadovska et al., 2015; Wen et al., 2016; Wiklander et al., 2015) or exosome surface marker fusion reporter proteins. Both of these approaches suffer from limitations regarding the reliability and quantifiability of the signal. For instance, membrane‐bound dyes like DiD, DiI, or PKH offer the flexibility of staining EVs without cell engineering, but the dye is transferable to other structures in vivo and persists well after the EVs have been metabolized (Gupta et al., 2020; Rieck, 2003). Fusion reporters are not incorporated into all EVs; thus, the resultant signal is weak with limited tissue detection depth making in vivo quantification challenging (Gupta et al., 2020). Measuring distribution using EV‐associated RNA via qPCR target has been attempted in the past but has relied largely on miRs which are evolutionarily conserved across species and, given their high background in naïve tissue, severely limiting their utility. To this end, others have relied on global knockout models of miRs, including miR‐155 (Bala et al., 2015) to more reliably assay biodistribution. This approach relies largely on the modulation of targets of the miR rather than direct quantification of the miR itself.

In this report, we have developed and implemented a novel qPCR‐based molecular platform to monitor EV biodistribution in vivo and in vitro. DUST enables tracking the biodistribution of EVs without modifying the EVs or producer cells. This approach involves the identification of a species‐specific, EV‐associated non‐coding RNA, screening donor tissue background for similar species to guide specific primer development, and qPCR optimization. The result is a highly efficient, sensitive, and specific assay for quantifiably measuring native EV biodistribution at the level of particles per mg of tissue.

Our findings also demonstrate that CDC‐sEVs are rapidly taken up by tissue, namely the heart, liver, and kidney. Retention by the liver and kidney is supported by several reports in the literature using the various approaches listed earlier (Choi & Lee, 2016; Di Rocco et al., 2016). However, proportionally higher retention by cardiac tissue is a novel finding and may suggest preferential EV uptake by certain cells. Indeed, studies of enhanced EV uptake by similar tissue (even in xenogeneic contexts) have been reported including tumour EVs (Garofalo et al., 2019; Kooijmans et al., 2016). Here we used EVs purified using a low molecular weight cutoff (10 KDa). The presence of soluble proteins in an EV preparation may also influence the biodistribution profile compared to more pure formulations.

Recent estimates suggest that a majority of intravenously injected EVs have a half‐life of 7 min (Matsumoto et al., 2020). Thus, at 1‐h post‐injection, about 0.3% of the original dose remain (Matsumoto et al., 2020). Our results suggest that a majority of NT4 is retained 1 h post‐administration (74%; Figure 3f). This discrepancy can be rationalized by two principal considerations. Firstly, the technique we describe here measures the retention of EV cargo rather than the intact EV itself. This is because there we cannot separate detection of NT4 inside EVs from the NT4 from EVs that have been uptaken by cells. Most EV biodistribution techniques rely on optical methods that mark the membrane of the EV not the cargo. So, it is conceivable that RNA cargo like NT4 sustains (through uptake by cells) long after the lipid bilayer particle is degraded. This is further supported by the observation that NT4 is no longer detectable when EV uptake is disrupted (Figure 5b). For this reason, it is not possible to measure EV half‐life using DUST. This method can, however, evaluate the half‐life of EV cargo like NT4 if retention is measured at multiple time points. As this is a proof‐of‐concept study, however, we focused only on a single timepoint and therefore only retention, not half‐life, was evaluated. There are strengths and drawbacks to optical tracking of EVs versus qPCR‐tracing of its RNA cargo. DUST is especially useful when evaluating the pharmacokinetics of therapeutic payload in EV therapy. Ultimately a comprehensive assessment of EV biodistribution would rely on both complimentary approaches in assessing EV signalling in vivo.

We further found that cell modification, site of intravenous administration, and injury all impact biodistribution. For instance, retro‐orbital administration retained more EVs in the brain. Finally, cardiac injury increased EV retention, particularly in the heart, specifically in the infarct area. Inhibition of EV surface docking to HSPGs inhibited CDC‐EV retention in all organs except the heart and kidney which points to a different mechanism by which cardiac tissue (or injured tissue in general) binds EVs for uptake. Inhibition of clathrin‐mediated endocytosis blocked EV retention across all organs. This demonstrates that EV retention in tissue is not driven by passive accumulation but an active cellular uptake mechanism. Inhibition of either of these critical pathways for EV uptake abrogated the therapeutic effect. It is interesting to note that despite EV retention in the heart, CDC‐EV administration was not cardioprotective which may point to a pleiotropic effect of systemically administered EVs. Intracardiac administration of CDC‐sEVs is cardioprotective so the effect here may be dose‐related. Finally, using this technique in vitro we demonstrate that macrophages, endothelial cells, and fibroblasts are the principal recipients of EVs compared to cardiomyocytes and thus cardioprotection is likely mediated by those cell types rather than a direct influence on the myocytes themselves. The variable uptake kinetics may potentially point to different predominating mechanisms of EV uptake (e.g., clathrin‐mediated endocytosis, caveolin‐mediated uptake, and macropinocytosis) among cell types (Kwok et al., 2021). Furthermore, the unique membrane composition (i.e., lipid rafts) of each cell type will impact fluidity which, in turn, influences uptake kinetics (Mulcahy et al., 2014). Finally, the observation that NT4 was detected primarily in the cytosol compared the nucleus suggests that bioactivity of this RNA may occur in this cellular compartment. It is important to note that using the current technique, we could not distinguish between free NT4 and NT4 sequestered by (and yet intact) lysosomes. Interestingly, the observed fluctuations of EV uptake over time could point to a “regurgitation effect” whereby cells re‐secrete uptaken cargo, a phenomena documented previously in the literature. Taken together, the findings confirm previous observations, and shed further light on EV biodistribution, enabling a more rigorous investigation of EV pharmacokinetics and pharmacodynamics.

Ultimately, this method is a proof of concept and outlines the parameters that could be used to identify suitable genes (from any RNA class with sufficient enrichment and specific‐specificity) to use as cargo tracers. This work provides crucial insight into how biodistribution changes with producer cell modification, different routes of administration, and injury.

Ultimately his method is not without its limitations. For instance, EVs from various cell types are enriched in a variety of RNA cargo some of which may not necessarily be species‐specific and thus limits the scope of utility for this approach. Furthermore, given the rapid clearance of EVs from circulation and tissue observed by us and others (Gupta et al., 2020; Imai et al., 2015), the sensitivity of DUST likely wanes significantly at later time points (more than 1 h post‐administration). Therefore, it will be important to identify the specific mechanisms by which EVs are cleared from the circulation (e.g., complement fixation, phagocytosis, etc.). Emerging methods are enabling more efficient retrieval of EVs from various biofluids (Chen et al., 2021), thus allowing further investigation of EV interaction with serum components that facilitate this rapid clearance. Furthermore, our technique is terminal as it depends on isolating RNA from the tissue. Taken together, DUST represents a proof‐of‐concept in assessing in vivo EV trafficking and signalling and further enables EV translation‐related efforts.

## DECLARATION OF INTEREST STATEMENT

Eduardo Marbán owns the founder's stock in Capricor Therapeutics. Alessandra Ciullo, Chang Li, Liang Li, Korie Candis Ungerleider, Kiel Peck, and Ahmed G.E. Ibrahim declare no competing interests.

## AUTHOR CONTRIBUTIONS

AC: conceived the idea, designed experiments, performed experiments, and data analysis, and wrote the manuscript. CL, LL: performed experiments and provided technical and design input. KP and UKC: performed experiments and data analysis. AGI and EM: conceived the idea, wrote the manuscript, and supervised the study.

## Supporting information



Supporting information.
