# Supplementary material for: Biodistribution of unmodified cardiosphere‐derived cell extracellular vesicles using single RNA tracing
Source: J Extracell Vesicles. 2022 Jan 10;11(1):e12178. doi: 10.1002/jev2.12178 (PMC8743874; doi:10.1002/jev2.12178)
Supplement: Supplementary file 1 — Supporting information. [file JEV2-11-e12178-s001.docx]

**Supplementary Figure 1: NT4 RNA is highly abundant in CDC-sEVs**

**a**

MFE= **-1.60** kcal/mol

**Centroid**

**secondary structure**

**MFE**

**secondary structure**

MFE= **-2.70** kcal/mol

Base-pair

probabilities

**b**

**c**

**d**


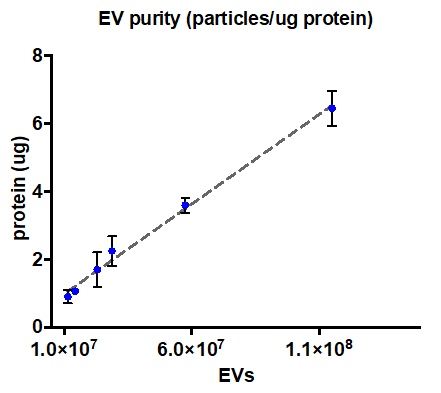

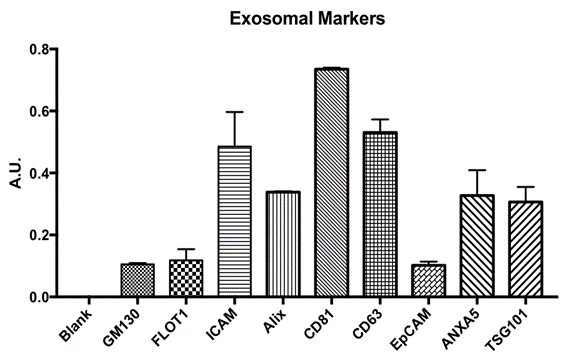

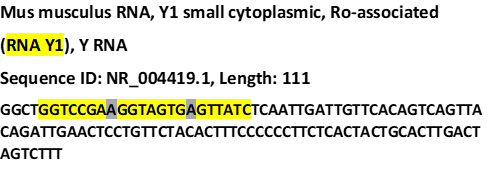

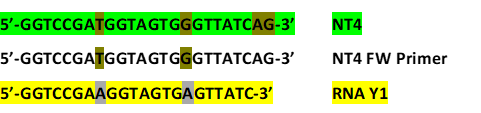

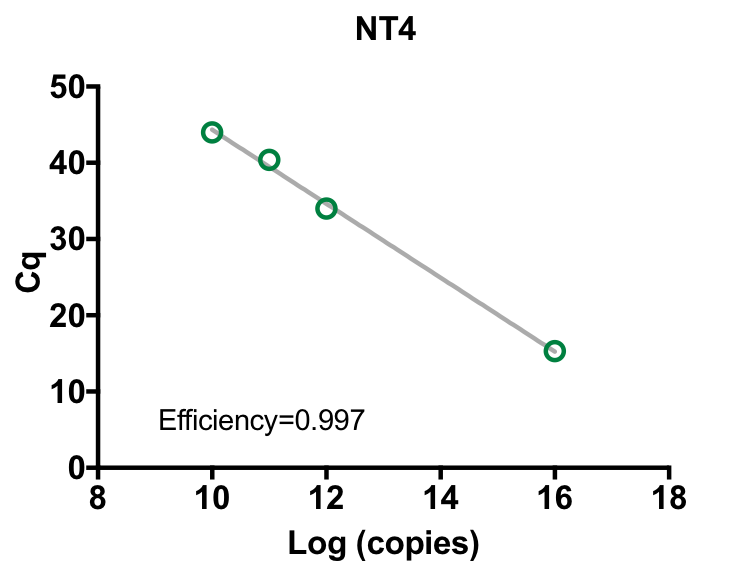


**e**

**f**

**Supplementary Figure 2: NT4 copy number in organs, plasma and urine**

c


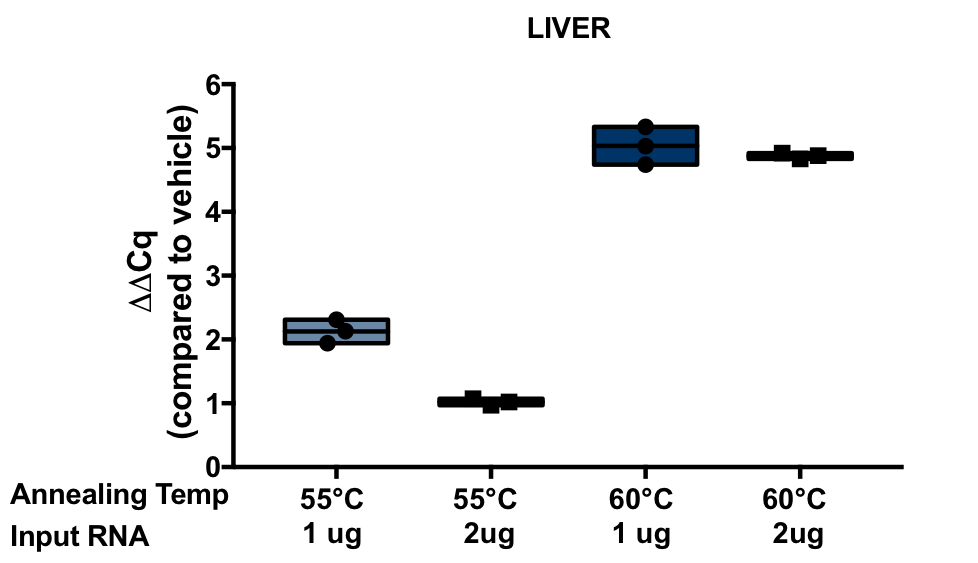

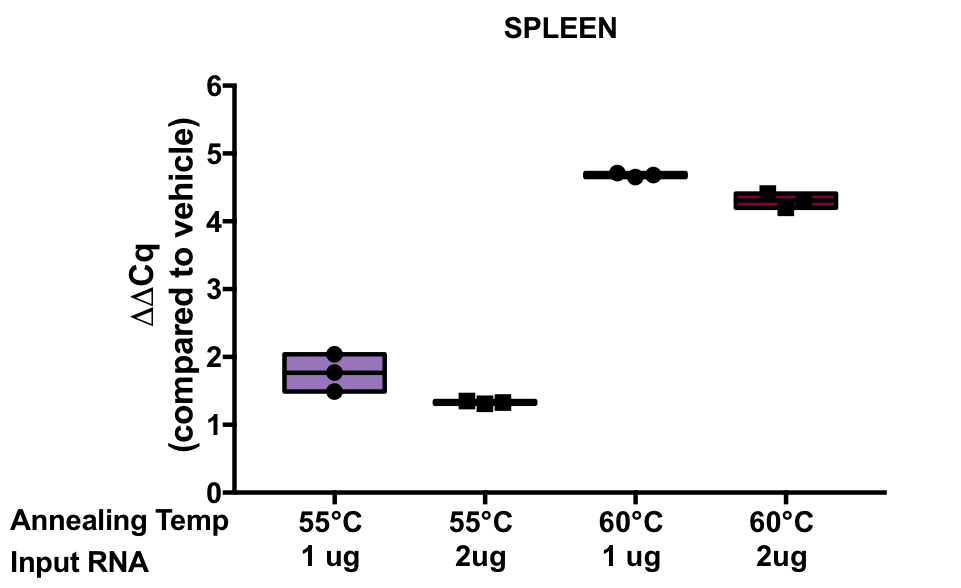


a

b

d

e

f

g

h

i

j

**a**

**b**

**Supplementary Figure 3: CDC-sEVs biodistribution in healthy animals after femoral vein injection (with prior perfusion) and CDC-sEVs isolated using SEC**


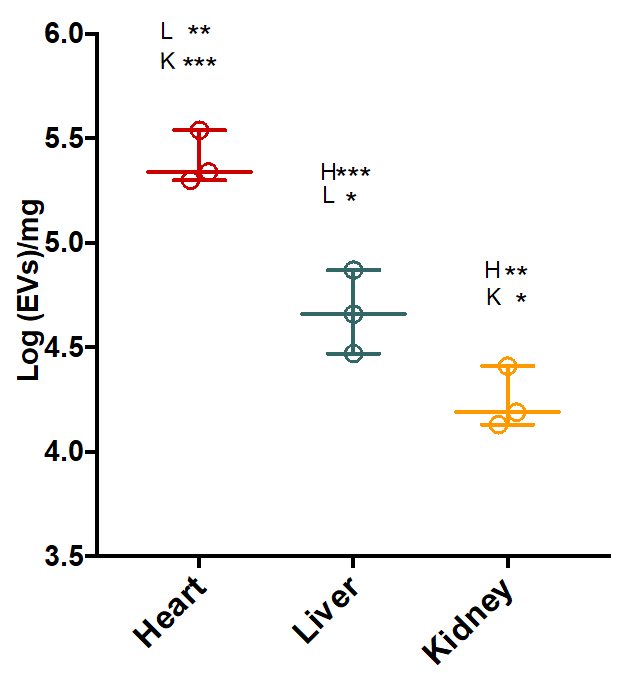

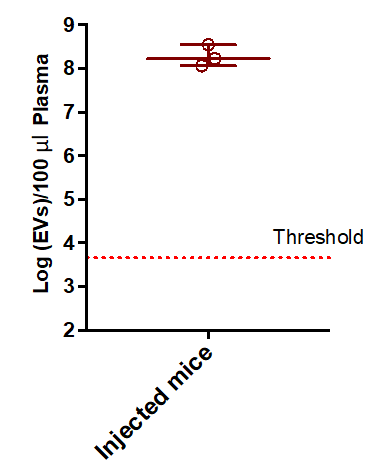

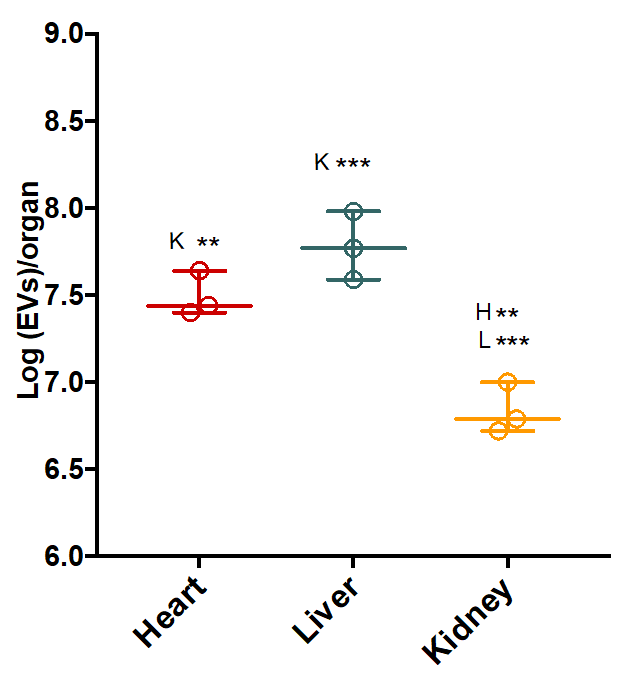


**c**

**d**

**e**

**Perfused organs**

**Perfused organs**

**SEC-purified sEVs**

**SEC-purified sEVs**

**SEC-purified sEVs**


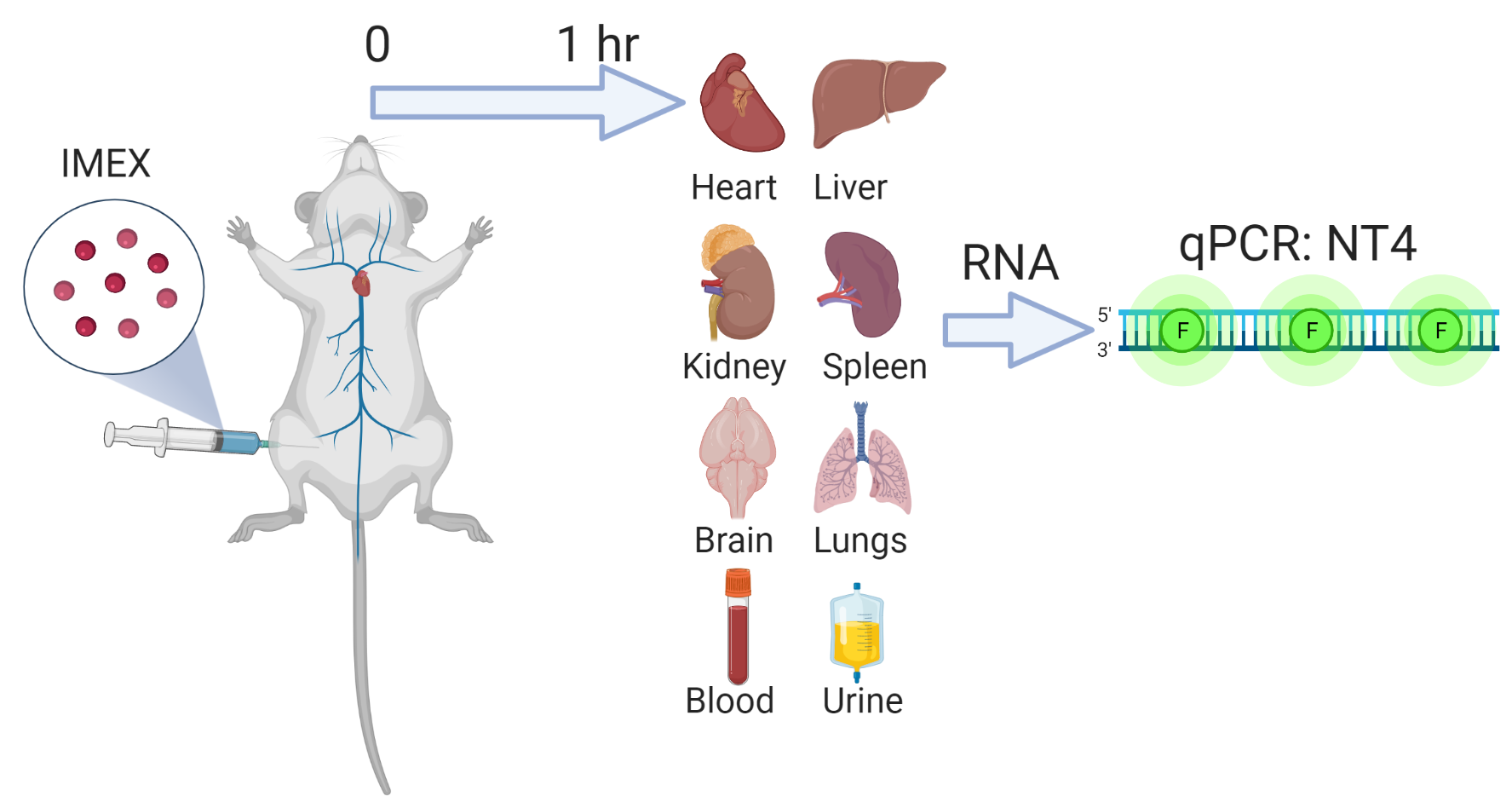


a

b

c

d

e

**Supplementary Figure 4: IMEX biodistribution in healthy animals after femoral vein injection**

**Supplementary Figure 5: CDC-sEVs biodistribution through retro-orbital administration**

a

b

d

e

g

c

f


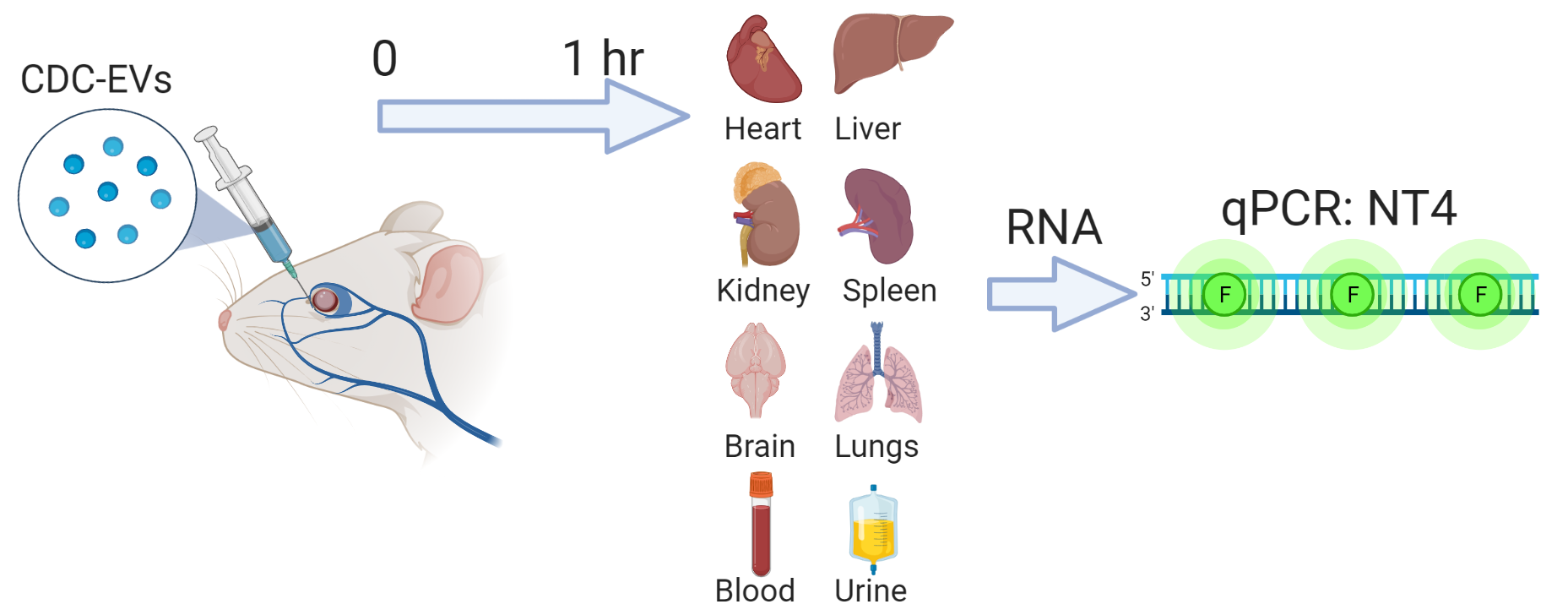


**Supplementary Figure 6: NT4 nuclear and cytosolic localization in macrophages**

a

**NT4**

***Supplementary Figure 1: NT4 RNA is highly abundant in CDC-sEVs***

(a) In silico analysis using an RNA structure prediction algorithm suggests that NT4 may assume two energetically probable stem-loop secondary structures characteristic of Y RNA molecules. (b) NCBI BLAST showing mouse RNA Y1, as 83% (20/24) identical to NT4. NT4 forward primer design excluded areas of high sequence identity with mouse RNA Y1. (c) qPCR for NT4 copy number calculation using known copies of NT4 (*n*=3 replicates per group; efficiency of NT4 qPCR was 0.997). (d) Purity measure comparing particle number per protein amount in serially diluted samples of EVs (*n*=3 replicates per group, mean ± SEM). (e) µg of proteins corresponding to the amount of CDC-sEVs and IMEX injected in animals for in vivo experiments (2x10^9^) (n=3-4 replicates per group; data presented as mean ± SEM). (f) Exosome protein markers were observed on CDC-sEVs using an Exosome protein array (*n*=2 replicates).

***Supplementary Figure 2:*** ***NT4 copy number in blood and urine***

(a,b) Optimization of qPCR protocol for NT4 amplification using different annealing temperatures (55°C and 60°C) and RNA input (used for reverse transcription). ΔΔCq was calculated by subtracting the NT4 ΔCq value of the treated sample to the NT4 ΔCq value of the vehicle. Both liver and spleen tissues were analyzed (n=3 biological replicates per group; data presented as ΔΔCq ± SEM). (c)The efficiency of NT4 amplification using known numbers of CDC-sEVs in plasma using ExoQuick® Exosome Isolation and RNA Purification Kit (for Serum & Plasma) (c) or Qiazol isolation method (d) and in urine (e) from healthy mice. (*n*=3 biological replicates per group; data presented as mean). (f-j) The efficiency of NT4 amplification using a standard curve prepared using serial dilutions of known numbers of CDC-sEVs spiked into 20 mg samples of homogenized mouse tissue (heart, kidneys, spleen, brain, lungs) (ΔCq is calculated by subtracting the U6 Cq value to the NT4 Cq value of the same sample).

***Supplementary Figure 3: CDC-sEVs biodistribution in healthy animals after femoral vein injection (with prior perfusion) and CDC-sEVs isolated using SEC***

Healthy mice received an intravenous (IV) injection of 2x10^9^ CDC-sEVs into the femoral vein. Animals were perfused prior organs collection. Heart, liver and kidneys were collected. (a) Biodistribution data in tissue one hour after administration of CDC-EV expressed in Log (EVs)/mg of tissue and whole organ (b). All data is presented as mean ± SEM (n=3 animals per group). (c) Healthy mice received an intravenous (IV) injection of 2x10^9^ CDC-sEVs isolated using size-exclusion chromatography (SEC) into the femoral vein. Animals were perfused prior organs collection. Heart, liver and kidneys were collected. (a) Biodistribution data in tissue one hour after administration of CDC-EV expressed in Log (EVs)/mg of tissue, whole organ (d) and plasma (e). All data is presented as mean ± SEM (n=3 animals per group, significance was determined using one-way analysis of variance with Tukey’s post-test with * = p < 0.05, ** = p < 0.01 and *** = p < 0.001).

***Supplementary Figure 4: IMEX biodistribution in healthy animals after femoral vein injection***

(a) Schematic of the experimental design. Healthy mice received an intravenous (IV) injection of 2x10^9^ IMEX or vehicle control (serum-free media) into the femoral vein. Heart, liver, kidneys, spleen, brain, and lungs were collected. IMEX distribution per mg of tissue (b), and whole organ (c) one-hour post-administration. (d) Percentage of total injected EVs detected in different organs (e) Difference in percentage distribution compared to CDC EV-treated animals. (n = 5 animals, data presented as mean ± SEM). Comparison between groups in (a-d) was evaluated using one-way ANOVA with Tukey’s. Comparisons between groups in (e) were made using Student’s T-test post-test. * = p < 0.05, ** = p < 0.01, *** = p < 0.001 and **** = p < 0.0001). Abbreviations above box plots reflect statistic differences between sample and other organ tissue; (B: brain, Lu: lung, H: heart, K: kidney, S: spleen, L: liver, P: Plasma, and U: urine).

***Supplementary Figure 5: CDC-sEVs biodistribution through retro-orbital administration***

(a)Schematic of the experimental design. Healthy mice received an intravenous (IV) injection of 2x10^9^ CDC-sEVs or vehicle control (serum-free media) into the retro-orbital venous sinus. Heart, liver, kidneys, spleen, brain, lungs, plasma, and urine were collected. CDC EVs per mg of tissue (b), whole organ (c), plasma (d), and urine (e), on hour post-retro-orbital administration. (f) Percentage of total injected EVs detected in different organs and (g) difference in percentage distribution using retro-orbital injection (RO inj) to CDC EV-treated animals using femoral vein injection (FV) (n= 4 animals per group; data presented as mean ± SEM). (a, b, c, and f) comparisons between groups were evaluated using a one-way ANOVA; (d, e, and g) comparisons between groups were evaluated using a Student’s T-test (* = p < 0.05, ** = p < 0.01, *** = p < 0.001 and **** = p < 0.0001). Abbreviations above box plots reflect statistic differences between sample and other organ tissue; (B: brain, Lu: lung, H: heart, K: kidney, S: spleen, L: liver, P: Plasma, and U: urine).

***Supplementary Figure 6: NT4 nuclear and cytosolic localization in macrophages***

(a)BMDM-Mø were treated with 80nM NT4 for 20 minutes and 24 hours. RNA was extracted from nuclear and cytosolic fractions and levels of NT4 were detected by qPCR (n=4 biological replicates per group; data presented as fold change(compared to vehicle), significance was determined using one-way analysis of variance with Tukey’s post-test with * = p < 0.05, and *** = p < 0.001).
